# Quebec Decision Rule in Determining the Need for Radiography in Reduction of Shoulder Dislocation; a Diagnostic Accuracy Study

**Published:** 2019-02-15

**Authors:** Ehsan Bolvardi, Behnaz Alizadeh, Mahdi Foroughian, Bita Abbasi, Seyed Reza Habibzadeh, Reza Akhavan

**Affiliations:** 1 Department of Emergency Medicine, Faculty of Medicine, Mashhad University of Medical sciences, Mashhad, Iran.; 2 Department of Radiology, Faculty of Medicine, Mashhad University of Medical sciences, Mashhad, Iran.

**Keywords:** Quebec, shoulder dislocation, decision support techniques, diagnostic imaging, radiography

## Abstract

**Introduction:**

The Quebec Decision Rule (QDR) has been developed for deciding on the necessity of radiography for patients with shoulder dislocation. This study aimed to investigate the diagnostic value of QDR in this regard.

**Method:**

This diagnostic accuracy study was conducted on patients with shoulder dislocation visiting the emergency department. After filling out the QDR-based checklist for all patients, they underwent radiography and the obtained radiography results were compared to QDR-based clinical diagnostic findings.

**Results:**

143 patients with the mean age of 32.1±12 years were evaluated (88.8% males). Sensitivity, specificity, and positive and negative predictive values of QDR were 50%, 58.2%, 3.3%, and 97.6%, respectively. The sensitivity and specificity were 100% and 50% in patients >40 years old, and 33.3% and 59.8% in those <40 years old. These indices were 33.3% and 60.4%, respectively, in the male sex and 100% and 40% in the female sex.

**Conclusion:**

**‌ **Quebec decision rule holds promise to diagnose concomitant fractures in patients over the age of 40 with 100% sensitivity, thereby reducing the number of radiographies by 50% without causing diagnostic errors. In contrast, this criterion proved inefficient in patients younger than 40. ‌

## Introduction

Shoulder dislocation is the most common type of joint dislocation in humans with the prevalence of 17-23 cases per each 100,000 population (1-3). Closed-reduction is usually a successful initial treatment for anterior shoulder dislocation (4). In the majority of cases, pre- and post-reduction radiography are advocated to confirm dislocation and ensure the complete reduction and evaluation for fractures (5).

Current studies have questioned the need for pre- and post-reduction radiographs in shoulder dislocation (5-7). According to researchers’ findings, radiographs are needed to confirm the location of the reduced joint in dislocations with an associated fracture or when the physician is uncertain (8). 

The Quebec Shoulder Dislocation (QSD) Rule is a clinical decision-making guideline, designed by a group of Canadian researchers, to guide physicians about the indications of radiography in patients with a shoulder dislocation. This guideline advocates the use of pre-reduction radiography in adult patients younger than 40 years if the mechanism of injury involves a motor vehicle collision, a fall from standing height, or a sports injury. In young people, these guidelines have a sensitivity of 100% and a negative predictive value of 99.2% in diagnosis of clinically important fractures and can reduce the number of pre-reduction radiographies by 27.9% (9). Nevertheless, all of these findings were not confirmed by all subsequent studies. 

Although these findings indicate that pre- and post-reduction radiography are not needed for all patients with shoulder dislocation, there is still no standard, reliable, generally applicable, and broadly accepted technique. Since the pre- and post-reduction radiography processes are significantly time-consuming and expose the patients to harmful radiation and impose a huge financial burden on them and the healthcare system, this study intended to investigate the diagnostic value of this guideline in patients with shoulder dislocation, visiting emergency department. ‌‌‌‌‌

## Method:

*Study design and setting*

This cross-sectional (diagnostic accuracy) study was conducted on patients with shoulder dislocation visiting Imam Reza and Shahid Hasheminejad Hospitals of Mashhad, Iran, between December 2016 and December 2017. This project was approved by the Ethics Committee of Mashhad University of Medical Sciences under the code IR.MUMS.fm.REC.1394.599. Researches adhered to principles of Helsinki declaration and confidentiality of patients’ information during the study period.

***Participants***

All patients who presented to the emergency departments of the mentioned hospitals during the study period were enrolled without any gender limitation using the convenience sampling technique. Patients less than 18 years old, as well as patients with neural or vascular injuries in the affected organ were excluded.

***Data gathering***

A checklist containing the baseline characteristics of the patients as well as QDR variables was designed for data gathering. After obtaining informed consent from the participants, the QDR-based designed checklist was completed for all participants. Then standard digital radiography (AP-lateral-Y view) was performed. If the radiographs confirmed dislocation, shoulder reduction would be carried out after procedural sedation and analgesia based on the existing department protocol (Fentanyl 1micro/kg + propofol 1mg/kg). Finally, results of QDR criteria regarding the need for imaging were compared to radiographs and its sensitivity, specificity, and negative and positive predictive values were calculated. Data Gathering and examinations were performed by emergency medicine physicians. All radiographs were interpreted by two emergency medicine physicians and a radiologist was consulted whenever there were disagreements or diagnostic uncertainties. 

According to QDR, pre-reduction radiography is necessary for adult patients younger than 40 if the injury involves a motor vehicle collision, fight, a fall from standing height, or sports injury. QDR also recommends radiography for patients under 40 with and without ecchymosis who have their first-time shoulder dislocation (10).

***Statistical analysis***

 The required sample size estimation was 140 patients based on the confidence interval of 95% and accuracy of 12% (10). The controlled data was fed into SPSS 12 and analyzed using tables, diagrams, and central and distribution indices. Findings were reported using mean ± standard deviation or frequency and percentage. P < 0.05 was considered statistically significant. Student’s t-test and chi square test were used for comparing the two groups regarding quantitative and qualitative variables, respectively. The diagnostic values including sensitivity, specificity, positive and negative predictive values, and positive and negative likelihood ratios were reported with 95% confidence interval. 

## Results

***Baseline characteristics of studied patients***

143 patients with the mean age of 32.1±12 (8 – 74) years were studied (88.8% male). The patients’ baseline characteristics, major causes of dislocation, side of dislocation, and pre-reduction radiographic findings are presented in table 1. Patients < 40 and > 40 years old were similar regarding the baseline characteristics. Patients < 40 years old had a significantly higher rate of recurrent dislocation (4.9±6.2 vs 1.5±0.5 times; p <0.001).

***Diagnostic accuracy of QDR***

According to QDR criteria, there was an indication for pre-reduction radiography in 60 (42%) patients. Two out of four patients (50%) with concomitant fractures had radiography indication based on QDR. The overall screening performance characteristics of QDR based on gender and age are presented in table 2. Quebec decision rule has an excellent sensitivity in patients older than 40 years, and also in females (both 100%). The observed positive likelihood ratio (LR+) for QDR criteria was 1.19 and the negative likelihood ratio (LR-) was 0.85 for the whole study population. However, the highest LR+ for this test belonged to patients older than 40 years and females.

## Discussion

Based on the QDR criteria, pre-reduction radiography was indicated in 42% of the study subjects. The sensitivities and specificities of QDR in diagnosis of concomitant fracture and need for pre-reduction were 50% and 58.2%, respectively. Application of QDR can reduce the number of radiographies by 56%. 

**Table 1:** Baseline characteristics of the study population

Variables	Age (years)		P value
	< 40 (n=120)	>40 (n=23)	
Gender			
**Male**	110 (91.7)	17 (73.9)	0.24
**Female**	10(8.3)	6(26.1)	
Type of dislocation			
**Anterior**	117 (97.5)	23 (100)	>0.99
**Posterior**	3 (2.5)	0 (0)	
Side of dislocation			
**Right**	92 (76.7)	19 (82.6)	0.78
**Left**	28 (23.3)	4 (17.4)	
Mechanism of dislocation			
**Motor vehicle collision**	13 (10.8)	0 (0)	N/A
**Fights**	8 (6.7)	0 (0)	
**Falling**	14 (11.7)	15 (65.2)	
**Exercise**	16 (13.3)	0 (0)	
**Other**	69 (57.5)	8 (34.8)	
Clinical signs			
**Ecchymosis**	3 (2.5)	0 (0)	>0.99
**None**	117 (97.5)	23 (100)	
Pre-reduction X-ray			
**Normal**	117 (97.5)	22 (95.7)	0.508
**Fracture**	3 (2.5)	1 (4.3)	
Post-reduction X-ray			
**Normal**	117 (100)	22 (100)	N/A
**Abnormal**	0 (0)	0 (0)	
Need for imaging (QDR)			
**Indicated**	48 (40.0)	12 (52.2)	0.357
**Not indicated**	72 (60.0)	11 (47.8)	

Data are presented as frequency (%). QDR: Quebec decision rule.

**Table 2:** Screening performance characteristics of Quebec decision rule in patients with shoulder dislocation

	Total	>40 years	< 40 years	Male	Female
Sen	50.0 (6.7-93.2)	100 (2.5-100)	33.3 (0.8-90.6)	33.3 (0.8-90.6)	100 (2.5-100)
Spec	58.2 (49.6-66.6)	50 (28.2-71.8)	59.8 (50.4-66.8)	60.4 (51.3-69.1)	40 (16.3-67.7)
PPV	3.3 (1.3-8.6)	8.3 (5.6-12.1)	2 (0.4-9.7)	2 (0.4-9.3)	10 (6.8-30.2)
NPV	97.6 (93.7-99.0)	100 (N/A)	97.2 (93.9-98.7)	97.4 (94.3-98.8)	100 (N/A)
LR+	1.19 (0.44-3.25)	2 (1.31-3.03)	0.83 (0.16-4.17)	0.84 (0.16-4.24)	1.66 (1.10-2.51)
LR-	0.85 (0.31-2.30)	0 (N/A)	1.11 (0.49-2.51)	1.10 (0.48-2.48)	0 (N/A)

Data are presented with 95 % confidence interval. Sen: Sensitivity; Spec: Specificity; PPV: Positive predictive value; NPV: Negative predictive value; LR+: Positive likelihood ratio; LR-: Negative likelihood ratio. N/A: not applicable.

Since pre- and post-reduction radiographies seem unnecessary for all patients with shoulder dislocation, a guideline for indication of radiography is needed in these patients. 

QDR is among the most important attempts made in this regard (10). Based on one study, in young people, QDR guideline has a sensitivity of 100% and a negative predictive value of 99.2% in diagnosis of clinically important fractures and can reduce the number of pre-reduction radiographies by 27.9% (10). Nevertheless, studies still cannot absolutely confirm or deny its efficiency in evaluation of shoulder dislocation.

Clinical evidence can lead experienced emergency medicine physicians to diagnosis of anterior shoulder dislocation and eliminate radiographies before reduction (11, 12). It has been reported that although 37% of fractures were observed in post-reduction radiographs, none of the missed cases affected the treatment outcome. Accordingly, although shoulder dislocation is likely to be associated with fractures, which may be missed in the initial radiographs because of their small size or joint position, they do not necessitate a different treatment process (13). 

Three important factors that correlated with fracture were age greater than 40 years, no prior dislocation, and injury mechanism. Accordingly, shoulder radiography is advocated for cases with a first-time dislocation, traumatic mechanism, and when the physician is uncertain of joint position. This algorithm reduced the number of radiographies by 46% and resulted in significant savings in time in a previous study. On the other hand, no missed fracture or dislocation was observed in cases without shoulder radiography (14).

There are some similar studies on the sensitivity and accuracy of QDR criteria. For example, Emond et al. (10) investigated the sensitivity and specificity of QDR criteria and found 100% sensitivity and 34.2% specificity, and a negative predictive value of 99.2%. The use of this criterion reduced the use of pre- and post-reduction radiographies by 27.9% and 81.9%, respectively.

 Abuye et al. (15) also investigated QDR and showed that this algorithm could reduce the number of radiographies by 50%. The researchers concluded that injury mechanism and recurrent dislocation are two important factors to determine whether pre-reduction radiography is needed. These findings are completely consistent with our findings.

Due to a significant difference in the mechanism of dislocation and the prevalence of associated fracture by age, we investigated the efficiency of QDR in two specific age groups (older and younger than 40). Results suggested a higher sensitivity and negative predictive value in patients older than 40 years. Ong et al. studied 196 patients under 40 and found that 59% of them were at high risk according to QDR. In total, 12 (6%) fracture cases were observed. Accordingly, QDR reached a sensitivity of 42%, a specificity of 40%, and a predictive value of 91% for associated fracture evaluation in such patients. 

Similar to the current study, Ong et al. showed inefficiency of QDR criteria in patients under 40 (5). Orloski et al. investigated 7,209 patients with dislocation, 465 (6.5%) of which had associated fracture. They found that a small percentage of fractures occurred in the second (0.7%) and third (0.8%) decades of life. The prevalence of fracture in the fourth and fifth decades of life was 2.6% and 4.6%, respectively. This rate increased by at least 19% in the 8th-10th decades of life. Not performing pre-reduction radiography for patients in their second and third decades of life may reduce pre-reduction radiography by 40%. According to them, since the risk of fracture is below 1% in the second and third decades of life, the use of routine pre-reduction radiography for patients with shoulder dislocation can be reduced, which is inconsistent with our findings (14).

 In our study, both LR+ and LR- in total population were similar with regard to the ability to differentiate patients who need radiography from those who do not. In other words, there is no difference in using this tool as a "screening" or "definite diagnosis" method for performing radiography before reduction. However, the highest LR+ was observed in females, it seems that it can be used as an acceptable "screening" tool in this gender. 

***Limitation***

In this study, sample size and hospital selection were among the limitations and studies with larger sample size and different hospitals are needed to confirm our results.

## Conclusion: 

QDR holds promise to accurately diagnose fracture-associated shoulder dislocation in patients above 40 years with a sensitivity of 100%. It can also reduce unnecessary radiographies in such patients by 50% without leaving any fracture undetected. 
